# Serum Spermidine in Relation to Risk of Stroke: A Multilevel Study

**DOI:** 10.3389/fnut.2022.843616

**Published:** 2022-04-07

**Authors:** Liqiang Zheng, Yanxia Xie, Zhaoqing Sun, Rui Zhang, Yanan Ma, Jiahui Xu, Jia Zheng, Qianyi Xu, Zhao Li, Xiaofan Guo, Guozhe Sun, Fuguo Xing, Yingxian Sun, Deliang Wen

**Affiliations:** ^1^School of Public Health, Shanghai Jiao Tong University School of Medicine, Shanghai, China; ^2^Department of Clinical Epidemiology, Shengjing Hospital of China Medical University, Shenyang, China; ^3^Department of Health Policy and Hospital Management, Shengjing Hospital of China Medical University, Shenyang, China; ^4^National Office for Maternal and Child Health Surveillance of China, West China Second University Hospital, Sichuan University, Chengdu, China; ^5^Department of Cardiology, Shengjing Hospital of China Medical University, Shenyang, China; ^6^College of Public Health, Shanghai University of Medicine and Health Sciences, Shanghai, China; ^7^Department of Biostatistics and Epidemiology, School of Public Health, China Medical University, Shenyang, China; ^8^Institute of Health Sciences, China Medical University, Shenyang, China; ^9^Department of Cardiology, The First Affiliated Hospital of China Medical University, Shenyang, China; ^10^Institute of Food Science and Technology, Chinese Academy of Agricultural Sciences, Beijing, China; ^11^Institute of Health Sciences, China Medical University, Shenyang, China

**Keywords:** spermidine, cardiovascular disease, nested case-control study, stroke, epidemiology

## Abstract

The relationship between serum spermidine levels and future cardiovascular disease risk has not yet been well elucidated in the general population based on community studies. Using a nested case-control study, we estimated the association between serum spermidine level and future stroke. New stroke cases had higher baseline levels of spermidine than controls [182.8 (141.8–231.5) vs. 152.0 (124.3–193.0), *P* < 0.001]. After multivariable adjustment, individuals with spermidine ≥ 205.9 nmol/L (T3) higher risks of stroke (HR 5.02, 95% CI 1.58–16.02) with the lowest quartile (< 136.9 nmol/L) as reference. The association between serum spermidine levels and risk of stroke seemed to be consistent and was reproducible in our cross-sectional studies. In addition, comparisons of the areas under receiver operator characteristics curves confirmed that a model including spermidine had better discrimination than without (0.755 vs. 0.715, *P* = 0.04). Here we report a close relationship exists between serum spermidine levels and risk of stroke.

## Introduction

Stroke is the second highest cause of death and the third highest cause of disability worldwide (1). In China, stroke was the third highest cause of death and was the leading cause of all-age disability-adjusted life years (DALYs) in 2017 (2). Although clinical factors assist in identifying most stroke cases, the underlying factors in a substantial number of cases remain unexplained (3). Therefore, identification of significant factors leading to stroke is important to prevent and manage stroke cases. Dietary factors that can influence cardiovascular health have been reported.

Spermidine is a polyamine present in our diet that is essential for the proper function of many metabolic processes. A part of the tissue polyamines has its origin in the diet or their production by the intestinal microbiome (4). Plant- and fungal-derived products are the most relevant sources of spermidine. In common human nutrition, whole grain products, vegetables and legumes are among the food groups with the highest spermidine content. Foods rich in spermidine are wheat germ (0.35 mg/g); soybeans (0.070–0.180 mg/g); select mushrooms (0.060-0.160 mg/g), such as almond mushroom (aka trumpet mushroom); and various nuts and seeds such as pine nuts (0.060 mg/g) (5, 6). Furthermore it is also involved in most cellular functions, including the activation of autophagy, DNA stability, transcription, translation, and apoptosis (7). In 2016, Eisenberg et al. demonstrated that spermidine supplementation can improve cardiovascular function as well as having beneficial effects on the health and lifespan of mice by enhancing cardiac autophagy (8). In humans, a high dietary spermidine intake correlates with reduced blood pressure (8), which is an important protective factor of stroke. Meanwhile, Soda et al. found that long-term enriched polyamine diets increase the blood polyamine level in humans (9). Based on these results, it was believed that serum spermidine has an inverse relationship with the risk of stroke.

However, several cross-sectional studies do not seem to support this inverse association; moreover, these studies indicated higher levels of serum spermidine were found in patients with stroke than in control subjects (10–13). However, these contradictory results have not been validated in prospective cohort studies, where this causal relationship may be further studied.

Therefore, we tested the association between serum spermidine levels and risk of future stroke using a nested case-control study from a prospective cohort study and a cross-sectional study of the community-based general population in China.

## Materials and Methods

### Study Population

The present study is based on two large-scale epidemiological survey conducted in rural areas of Liaoning province. One was a prospective cohort study and the other was a cross-sectional study. The nested case-control study from a prospective cohort design. From January 2012 to August 2012, 11,956 participants over 35 years of age from 3 towns (26 rural villages) were selected to evaluate the prevalence, incidence, and natural history of the risk factors for cardiovascular disease in rural areas of Liaoning province (14). All of these subjects were invited to attend the two follow-up visits from August 2015 to January 2016 and December 2017 to January 2018. Of the 11,956 subjects, 10,700 participants consented and qualified for our follow-up study. A total of 10,349 participants (96.7%) completed at least one follow-up visit. For the current analyses, we excluded participants with a baseline history of stroke (*n* = 387) and coronary heart disease (CHD, *n* = 520). Using the median follow-up time of 4.66 years, 342 new incident stroke cases were identified in this prospective cohort. Of those, 101 eligible stroke cases were randomly selected for our analysis. Utilizing risk-set sampling, we then randomly selected control subjects who remained stroke-free and matched them to patients with stroke in a 1:1 ratio, according to age (difference within 3 years), sex (male/female), duration of the follow-up period (within 1 month), and hypertension (yes/no). On the basis of these criteria, 101 new patients with stroke and 101 matched stroke-free control subjects were selected for present study.

Considering the small sample size of the nested case-control study, we added data from the cross-sectional study to support our study. Both studies are from one team, and the inclusion and exclusion criteria are basically the same. During June 2019 and August 2019, a questionnaire survey was conducted in the general population. 4689 participants with 35 years of age or older were recruited as study population. Among the population, 3283 participants in three towns were selected to measure serum spermidine. We further excluded participants missing information of confounding factors (*n* = 146). And participants with CHD were excluded (*n* = 492). Finally, 2645 participants were enrolled in the present study. Among them, patients with stroke were matched to those without stroke approach 1:2 by nearest neighbor matching on propensity scores with a caliper of 0.2. Variables thought to be potential confounders were entered into the propensity score and included sex, age and history of hypertension. Finally, 221 patients with stroke and 442 matched stroke-free control subjects were selected for present study.

We defined CHD as self-reported physician diagnosis, including angina pectoris (ICD-10 code I20), acute myocardial infarction (AMI, I21), subsequent myocardial infarction (I22), other forms of acute (I24) or chronic (I25) heart disease and percutaneous transluminal coronary angioplasty or coronary artery bypass graft (15, 16).

### Detection of Spermidine in Serum Using High-Performance Liquid Chromatography With Fluorescence Detection

#### Chemicals and Reagents

Spermidine trihydrochloride (SPD) was obtained from Dr. Ehrenstorfer (Augsburg, Germany). The internal standard (IS), 1,7-diaminoheptane, was supplied by Sigma-Aldrich (Steinheim, Germany). Dansyl chloride from Solarbio (Beijing, China) was used as the derivatization reagent. The HPLC-grade acetonitrile (ACN) was purchased from Fisher (Waltham, MA, United States). Deionized water was obtained from Wahaha (Hangzhou, China). All other reagents were of analytical grade; 0.1 M sodium carbonate buffer (Na_2_CO_3_-NaHCO_3_ buffer) was prepared by mixing 0.1 M sodium carbonate and 0.1 M sodium bicarbonate (3:7 v/v) to a final pH 10; 0.1 M hydrochloric acid (HCl) was prepared by diluting 36% HCl (Shanghai, China).

#### Standard Solutions

The spermidine trihydrochloride (SPD) stock solution was prepared in 0.1 M HCl at a concentration of 1 mg/mL and stored at 4°C. A series of SPD standard solutions were prepared by diluting stock solution with 0.1 M HCl to get the concentrations 3–100 μg/L. The 5 mg/mL dansyl chloride standard solution was made in acetone and stored at 4°C.

#### Detection of Spermidine

The spermidine in serum was detected by the HPLC method described with some modifications. Briefly, 200 μL 0.1 M HCl was added to 100 μL serum, followed by 3 min of vortex-mixing to transform spermidine into SPD. Then 1 mL ACN was added to the mixture to precipitate the serum protein. After vortex-mixing for 2 min, the mixture was then centrifuged at 12,000 × *g* for 10 min. The supernatant was transferred into a 10 mL EP tube and dried *via* evaporation in a water bath (45°C) under a gentle stream of nitrogen gas. Then 200 μL of 0.1 M HCl was added to dissolve the dry residue. The 200 μL solution was then collected for derivatization using dansyl chloride as described. After derivatization, 20 μL aliquots of dansyl derivatives were injected into the HPLC system for analysis. The mobile phase solutions A and B were ultra-pure water and ACN, respectively. Gradient elution was selected at 0–7 min, 55–50% A; 7–25 min, 50–10% A; 25–31 min, 10% A; 31–35 min 10-55% A; 35–40 min 55% A. Other detection conditions remained unchanged.

### Study Outcomes

Based on the WHO Multinational Monitoring of Trends and Determinants in Cardiovascular Disease criteria (17), stroke was defined as a condition with rapidly developing signs of focal (or global) disturbance of cerebral function lasting > 24 h (unless interrupted by surgery or death) with no apparent non-vascular cause, including ischemic stroke and hemorrhage stroke. Transient ischemic attacks and silent brain infarctions (cases without clinical symptoms or signs) were not included, neither were events associated with trauma, hematologic disorders, or malignancy. The information was obtained by a single investigator’s direct reference to medical records. All materials were independently reviewed by the end-point assessment committee, whose members were blinded to the study participants’ baseline risk factor information.

### Data Collection and Physical Examinations at Baseline

The detailed methodology on data collection and physical examination is similar to our previous studies (18–20). At baseline examination, all participants were recruited and examined in a single clinic visit by their local doctors. A standard epidemiological questionnaire was used to collect data on demographic variables (age, sex, and ethnicity), smoking status, use of alcohol, information on antihypertensive medications, and if they had a history of stroke and CHD. Blood samples were collected after they fasted for 12 h. Total cholesterol (TC), triglyceride (TG), low-density lipoprotein cholesterol (LDL-C), and high-density lipoprotein cholesterol (HDL-C) were measured using automated enzymatic procedures. Systolic blood pressure (SBP) and diastolic blood pressure (DBP) were measured using a standardized automatic electronic sphygmomanometer (HEM-907; Omron, Tokyo, Japan).

### Statistical Analyses

The continuous variables were taken as the mean ± standard deviation (SD) and compared using the Student’s *t*-test or Mann-Whitney U test. The categorical variables for independent proportions were expressed as the ratio of frequency and Pearson’s χ^2^-tests.

In the nested case-control study, the following analyses were performed: we first observed the univariate relationship between different percentile levels of spermidine and stroke using Kaplan-Meier survival estimates and the log-rank test. In this step, we temporarily ignored our research type, a nested case-control study, with the purpose of proving whether spermidine may be associated with stroke. Before further multivariable analysis, we tested multicollinearity by incorporating all the preliminary variables into the ordinary least squares (OLS) model (21). The incidence-density sampling method was used to match controls to case patients on the basis of the cohort person-time, while conditional logistic regression models were used to examine the associations between the baseline concentrations of spermidine and the risk of stroke. The findings from the two models were reported. Model 1 presented the crude odds ratios (ORs) and 95% confidence intervals (CIs), which considered the ethnicity and baseline age. Model 2 further provided adjusted ORs and 95% CIs after the model was attuned for (i) ethnicity and baseline age; (ii) baseline blood pressure, including SBP and DBP; (iii) the subject’s baseline body mass index (BMI), TC, TG, LDL-C, HDL-C; and (iv) other major risk factors associated with stroke, such as smoking, drinking, and anti-hypertensive medications. In these models, spermidine enters the model as a continuous variable (10 nmol/L) or as tertiles (T3, ≥ 205.9 nmol/L; T2, 172.5–205.9 nmol/L; T1, < 136.9 nmol/L), with the lowest tertile (T1) as the reference group. In addition, receiver operating characteristic (ROC) curves were constructed, and the areas under the curves (AUC) were calculated to assess the discriminant power of spermidine for stroke incidence. Furthermore, we have performed various subgroup analyses, such as in regard to sex, and for an aging population (≥ 65 years old), moreover, we have even excluded the cases and their controls whose stroke occurred within one year after the follow-up for sensitivity analysis.

Because we noted important differences in baseline clinical characteristics between stroke and non-stroke, we did a second analysis based on propensity scores using cross-sectional data to mitigate confounding bias caused by the imbalance between the characteristics of the groups under comparison. Patients with stroke were matched to those without stroke approach 1:2 by nearest neighbor matching on propensity scores with a caliper of 0.2 (without replacement). To obtain the score, we fitted a logistic regression model, with stroke as a binary dependent variable and adjusted with the same covariates used in the nested case-control matching process (sex, age and history of hypertension). After matching, we compared serum spermidine levels between stroke and those non-stroke as matched groups. We calculated adjusted ORs and 95% CIs to quantify the association between serum spermidine levels and stroke, and used logistic regression fitted by generalized estimating equations to account for matched data. In these models, spermidine enters the model as a continuous variable (10 nmol/L) or as tertiles (T3, ≥ 111.0 nmol/L; T2, 48.0–111.0 nmol/L; T1, < 48.0 nmol/L), with the lowest tertile (T1) as the reference group.

All statistical calculations were performed using IBM SPSS statistical software version 23.0 (SPSS Inc., Chicago, IL, United States), R 4.1.1, and Stata software, version 16.0 (Stata Corp LP., College Station, United States of America). A 2-side *P* value of < 0.05 was considered statistically significant.

## Results

Individuals who had stroke during the follow-up period were more likely than their controls to have a higher level of SBP, DBP, and TG and a lower level of HDL-C (*P* < 0.05), whereas no statistically significant difference in BMI, LDL-C, fasting glucose, ethnicity, smoking history, and alcohol history between case and control groups (Table 1). In those the individuals were taking more antihypertensive medications than their controls (42.6% vs. 25.7%, *P* = 0.017). Similar characteristics were also observed in cross-sectional study. As a result of the matching criteria, the variables for age, sex and hypertension proportion were identical in the two groups.

**TABLE 1:** Characteristics of stroke patients and controls in prospective and retrospective case-control study.

	Nested case-control study			Cross-sectional study		
	Stroke cases	Controls	*P-*value	Stroke cases	Controls	*P-*value
*N*	101	101		221	442	
Sex, men/women	67/34	67/34	–	116/105	233/209	0.96
Age, yrs	62.7 ± 8.7	62.3 ± 8.5	0.73	64.2 ± 7.6	64.3 ± 7.6	0.89
Ethnicity, Han/Others	Nov-90	Apr-97	0.11	156/65	299/143	0.44
Hypertension, yes/no	81/20	81/20	–	172/49	342/100	0.9
SBP (mmHg)	160.8 ± 27.2	150.7 ± 19.8	0.003	145.9 ± 20.6	146.9 ± 19.8	0.55
DBP (mmHg)	89.2 ± 15.1	84.1 ± 9.9	0.005	85.2 ± 10.7	83.9 ± 10.5	0.12
BMI (Kg/m^2^)	24.8 ± 3.3	25.2 ± 3.3	0.39	25.2 ± 3.5	25.0 ± 3.6	0.48
TC (mmol/L)	5.5 ± 1.2	5.4 ± 0.9	0.63	5.2 ± 1.1	5.3 ± 0.9	0.13
TG (mmol/L)	1.8 ± 1.2	1.4 ± 0.9	0.03	1.7 ± 1.7	1.7 ± 1.7	0.59
HDL-C (mmol/L)	1.3 ± 0.4	1.45 ± 0.5	0.01	1.1 ± 0.4	1.2 ± 0.3	0.003
LDL-C (mmol/L)	3.2 ± 0.9	3.1 ± 0.7	0.59	3.3 ± 0.9	3.3 ± 0.8	0.66
Glucose (mmol/L)	6.31 ± 2.2	6.0 ± 1.1	0.14	6.5 ± 2.6	6.0 ± 1.8	0.004
Current smoking, n (%)	48 (47.5)	39 (38.6)	0.26	107 (48.4)	199 (45.0)	0.41
Current drinking, n (%)	26 (25.7)	29 (28.7)	0.75	89 (40.3)	185 (41.9)	0.70
Antihypertensive medications, n (%)	43 (42.6)	26 (25.7)	0.02	114 (51.6)	106 (24.0)	< 0.001
Spermidine(nmol/L)	182.8 (141.8–231.5)	152.0 (124.3–193.0)	< 0.001	146.4 (77.4–289.6)	54.4 (32.4–95.4)	< 0.001

*SBP, systolic blood pressure; DBP, diastolic blood pressure; BMI, body mass index; TC, total cholesterol; TG, triglycerides; HDL-C, high-density lipoprotein cholesterol; LDL-C, low-density lipoprotein cholesterol.*

**FIGURE 1:**
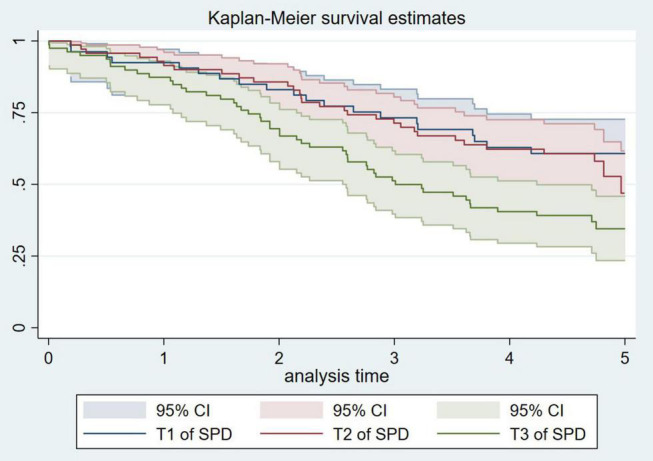
Kaplan–Meier curves for incident stroke according to the tertiles of serum spermidine of nested case-control study.

Using the HPLC with fluorescence detection (FLD) assays, spermidine was detected in all the study subjects. Participants who with stroke had higher levels of spermidine than in their control group [the prospective nested case-control study: median (inter-quartile): 182.8 (141.8–231.5) nmol/L vs. 152.0 (124.3–193.0) nmol/L, *P* < 0.001; the cross-sectional study: median (inter-quartile): 146.4 (77.4–289.6) nmol/L vs. 54.4 (32.4–95.4) nmol/L, *P* < 0.001]. Additionally, we observed a higher risk of stroke incidence with an increasing baseline serum spermidine concentration from the prospective nested case-control study (Figure 1).

**FIGURE 2:**
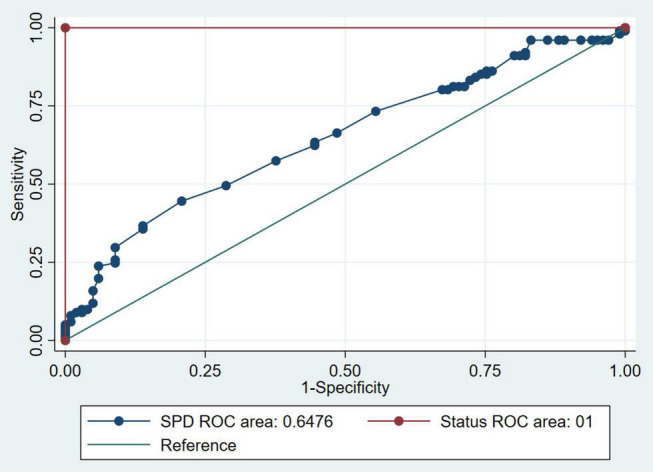
Receiving operating characteristics curves for incident stroke from SPD of nested case-control study.

As shown in Table 2, every 10 nmol/L increase in serum spermidine increased the risk of stroke by 11% (OR 1.11, 95% CI 1.05–1.18), and participants with spermidine levels over 205.9 nmol/L (T3) had OR for stroke of 3.70 (95% CI: 1.55–8.84) after adjusting for age and ethnicity among nested-case control study. Similar results were observed after further adjustment for other potential confounders, where for each 10 nmol/L increase in serum spermidine, the risk of stroke increased by 13% (OR 1.13, 95% CI 1.04–1.23). Compared with participants with spermidine less than 136.9 nmol/L, participants with more than 205.9 nmol/L had higher risks of stroke (OR 5.02, 95% CI 1.58–16.02). Furthermore, after we excluded the population that had stroke within one year, and the corresponding control group, we still obtained similar results. Propensity score regression analysis using cross-sectional data showed an association between serum spermidine and risk of stroke, which for each 10 nmol/L increase in serum spermidine the risk of stroke increased by 6% (OR 1.06, 95% CI 1.04–1.08). Overall, the highest serum spermidine level faced higher risks of stroke. The ROC curves only for risk of stroke and SPD had an AUC of 0.65 (95% CI:0.57∼0.72) (Figure 2).

**TABLE 2:** Relationship between serum spermidine levels and stroke in prospective and retrospective case-control study.

	Model 1		Model 2	
	HR (95% CI)	*P-value*	aHR (95% CI)	*P-*value
**Conditional logistic regression models †**				
SPD_10	1.11 (1.05,1.18)	0.001	1.13 (1.04,1.23)	0.003
Tertiles of SPD				
T1	1.00 (Ref.)		1.00 (Ref.)	
T2	1.45 (0.63,3.35)	0.386	2.80 (0.89,8.87)	0.079
T3	3.70 (1.55,8.84)	0.003	5.02 (1.58,16.02)	0.006
**Excluding the population with stroke within one year and the corresponding control:**				
SPD_10	1.10 (1.03,1.17)	0.004	1.12 (1.02,1.22)	0.014
Tertiles of SPD				
T1	1.00 (Ref.)		1.00 (Ref.)	
T2	1.02 (0.41,2.52)	0.964	2.00 (0.56,7.10)	0.287
T3	2.91 (1.16,7.29)	0.023	4.02 (1.13,14.36)	0.032
**Propensity score regression‡**				
SPD_10	1.06 (1.04, 1.08)	< 0.001	1.060 (1.04, 1.08)	< 0.001
Tertiles of SPD				
T1	1.00 (ref.)		1.00 (ref.)	
T2	2.35 (1.43, 3.86)	0.001	2.219 (1.31, 3.75)	0.003
T3	12.02 (7.34, 19.69)	< 0.001	12.794 (7.50, 21.82)	< 0.001

*† Data from the nested case-control study; ‡Data from the cross-sectional study; CI: indicates confidence interval; HR; hazard ratio. Model 1, adjusted for age, ethnicity. Model 2, adjusted for age, ethnicity, SBP, DBP, BMI, TC, TG, LDL-C, HDL-C, current smoking, current drinking, history of hypertension, anti-hypertensive medications.*

Several sensitivity analyses have been conducted as Table 3 shown. The association between spermidine and stroke incidence have been observed in males, and in participants older than 60 years in prospective nested case-control studies. Data from cross-sectional studies show that the relationship between spermidine and risk of stroke exists across different age and sex groups.

**TABLE 3:** Relationship between serum spermidine levels and stroke risk stratified by sex/age groups in prospective and retrospective case-control study.

	Model 1		Model 2	
	HR (95% CI)	*P-value*	aHR (95% CI)	*P*-value
**Conditional logistic regression models †**				
*Male*				
SPD_10	1.07 (1.01,1.14)	0.028	1.14 (1.02,1.26)	0.018
Tertiles of SPD				
T1	1.00 (Ref.)		1.00 (Ref.)	
T2	1.00 (0.37,2.75)	0.993	2.63 (0.53,13.09)	0.236
T3	2.10 (0.76,5.77)	0.152	5.01 (1.07,23.4)	0.041
*Female*				
SPD_10	1.30 (1.08,1.57)	0.007	1.01 (0.34,3.01)	0.981
Tertiles of SPD				
T1	1.00 (Ref.)		1.00 (Ref.)	
T2	2.82 (0.53,15.04)	0.224	–	0.593
T3	13.14 (1.79,96.43)	0.011	–	0.577
< *60 y*				
SPD_10	1.10 (0.99,1.23)	0.082	1.01 (0.46,2.21)	0.977
Tertiles of SPD				
T1	1.00 (Ref.)		1.00 (Ref.)	
T2	1.51 (0.40,5.63)	0.542	–	0.953
T3	4.47 (0.77,26.07)	0.096	–	0.986
≥ *60 y*				
SPD_10	1.11 (1.02,1.20)	0.011	1.16 (1.01,1.32)	0.035
Tertiles of SPD				
T1	1.00 (Ref.)		1.00 (Ref.)	
T2	2.06 (0.60,7.08)	0.250	3.72 (0.56, 24.89)	0.175
T3	3.68 (1.23,11.05)	0.020	8.33 (1.29,53.83)	0.026
**Propensity score regression‡**				
*Male*				
SPD_10	1.03 (1.02, 1.05)	< 0.001	1.04 (1.02, 1.05)	< 0.001
Tertiles of SPD				
T1	1.00 (ref.)		1.00 (ref.)	
T2	2.51 (1.29, 4.88)	0.007	2.74 (1.32, 5.68)	0.007
T3	8.89 (4.58, 17.25)	< 0.001	11.554 (5.38, 24.81)	< 0.001
*Female*				
SPD_10	1.24 (1.17, 1.32)	< 0.001	1.250 (1.17, 1.34)	< 0.001
Tertiles of SPD				
T1	1.00 (ref.)		1.00 (ref.)	
T2	2.16 (0.99, 4.71)	0.053	1.93 (0.84, 4.41)	0.122
T3	21.54 (9.90, 46.87)	< 0.001	23.31 (9.95, 54.60)	< 0.001
< *60 y*				
SPD_10	1.04 (1.01, 1.07)	0.003	1.04 (1.01, 1.06)	0.007
Tertiles of SPD				
T1	1.00 (ref.)		1.00 (ref.)	
T2	1.94 (0.71, 5.25)	0.194	2.81 (0.88, 9.01)	0.820
T3	12.89 (4.81, 34.57)	< 0.001	22.00 (6.39, 75.71)	< 0.001
≥ *60 y*				
SPD_10	1.07 (1.05, 1.10)	< 0.001	1.07 (1.05, 1.10)	< 0.001
Tertiles of SPD				
T1	1.00 (ref.)		1.00 (ref.)	
T2	2.47 (1.37, 4.44)	0.003	2.95 (1.55, 5.63)	0.001
T3	12.67 (7.08, 22.66)	< 0.001	16.78 (8.61, 32.70)	< 0.001

*† Data from the nested case-control study; ‡Data from the cross-sectional study; CI: indicates confidence interval; HR; hazard ratio.*

*Model 1, adjusted for age, ethnicity.*

*Model 2, adjusted for age, ethnicity, SBP, DBP, BMI, TC, TG, LDL-C, HDL-C, current smoking, current drinking, history of hypertension, anti-hypertensive medications.*

**FIGURE 3:**
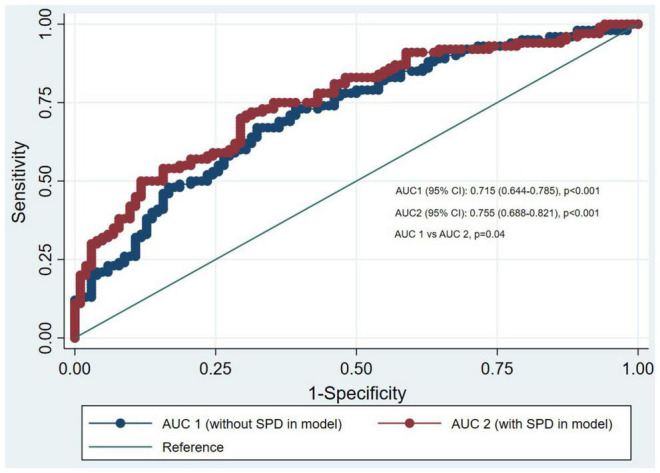
Receiving operating characteristics curves for incident stroke from multivariable conditional logistic regression models with and without spermidine of nested case-control study.

Figure 3 shows the ROC curves for risk of stroke incidence from multivariable conditional logistic regression models, with and without spermidine among the prospective nested case-control study. The end points were characterized with stroke, thus, the first model incorporating age, ethnicity, the status of baseline hypertension, SBP, DBP, BMI, GLU, TC, TG, HDL-C, LDL-C, smoking history, alcohol history, and antihypertensive medications had an AUC of 0.715 (95% CI: 0.644–0.785). The second model was supplemented with spermidine and led to a significant increase (*P* = 0.04) in the fit of the model [AUC was 0.755 (95% CI: 0.688–0.821)].

## Discussion

At present, the relationship between serum spermidine levels and future CVD risk has not been well characterized in the general population. In this study, we first used the HPLC-FLD assays to develop a novel stroke prediction model by detecting spermidine. We found that new stroke cases had higher baseline levels of spermidine than that in controls. After multivariable adjustment, the association between serum spermidine levels and risk of stroke seemed to be consistent and was reproducible among conditional logistic and propensity score regression models. Moreover, the relationship between serum spermidine and stroke was significant only in males or in individuals older than 60 in nested case-control study, while its association was significant across sex and age groups in cross-sectional study. Additionally, comparisons of the areas under the receiver operator characteristics curves confirmed that a model including spermidine had a better discrimination than one without (0.755 vs. 0.715, *P* = 0.04).

At present, most studies on spermidine and cardiovascular diseases focus on dietary spermidine (8, 22–24). Results from previous epidemiological studies corroborate that the intake of dietary spermidine (or that of spermidine and spermine combined) inversely correlates with the incidence of CVD and death in the Bruneck cohort (8). A cross-sectional regression meta-analysis of nutritional polyamine content alongside CVD-derived mortality rates from 48 western countries identified a negative association between spermidine and CVD (25). Most of these studies based on animal models confirmed these conclusions. Thomas et al. reported that spermidine may reverse age-induced arterial stiffness by reducing oxidative damage in the endothelial cells of aged mice (8, 26–29). In addition, in Dahl salt-sensitive rats fed with a high-salt diet (a model of hypertensive heart failure), oral supplementation of spermidine reduced high blood pressure and delayed the transition to heart failure (8), further documenting the antihypertensive (29) and vascular health-promoting (27, 28) functions of dietary spermidine.

However, few studies have shown the same relationship between serum spermidine levels and CVD or stroke. Therefore, our study highlights novel information on the pathogenesis of these diseases. In the present study, an association between serum spermidine and stroke was observed in a cross-sectional study. It was further confirmed in a prospective study that the baseline serum spermidine level is related to the incidence of stroke, and may be a predictor of the stroke incidence, although the sample size is relatively small. For each 10 nmol/L increase inserum spermidine, the risk of stroke increased by 13% (OR 1.13, 95% CI 1.04–1.23). Compared with the participants with spermidine less than 136.9 nmol/L, those with levels above 205.9 nmol/L had higher risks of stroke (OR 5.02, 95% CI 1.58–16.02). However, emerging evidence propose that oral SPD has a number of beneficial effects in human health. It may indicate that increased levels of Spd could be due to certain pathological conditions and/or aging. An *in vivo* study showed that the injection of spermidine into the ventricle or carotid artery can destroy the integrity of the blood-brain barrier within 15 min (30, 31). This result provides a possible mechanistic explanation for our findings. Elevated levels of spermidine in the bloodserum may destroy the blood-brain barrier, causing exogenous risk factors, that were originally blocked from entering the ventricle, to permeate into the ventricle through the damaged blood-brain barrier, thus increasing the risk of stroke. Spermidine can generate the metabolite acrolein through redox reactions (30). Acetylpolyamine oxidase and spermine oxidase play an important role in this process (13). Current studies on the relationship between these two enzymes and the occurrence of stroke have shown that their blood levels increase significantly in the early stages of stroke; therefore, a large amount of spermidine is oxidized to acrolein (13). This short-term downregulation of spermidine may activate a compensatory mechanism in the body, resulting in an increase in the level of spermidine entering the blood *via* this mechanism. Acrolein is from a class of substances that are proven to be cytotoxic to nerve cells (32). Although there is a positive correlation between spermidine levels and stroke, there is a possibility of superposition of these two mechanisms. The compensatory increase of spermidine further destroys the blood-brain barrier, which in turn increases the chance of acrolein and other harmful substances to enter the ventricle, thus increasing the risk of stroke. Another possible explanation is that geroprotective effects of spermidine in influencing lifespan extension in a hormetic dose response manner (33). In this hormesis scenario, a high dose of natural compounds is detrimental or toxic because inhibits antioxidant stress response pathways, while a low dose elicits a protective stress response in the organism. It is consistent with our finding that higher serum levels of spermidine lead to greater probabilities of adverse effects and increase stroke incidence compared to controls. Taken together, these results suggest that future studies should target the levels of the spermidine oxidation product in the blood and dynamically observe the changes to spermidine in the blood to verify the mechanism.

The main strengths of this study are as follows: (i) this is the first study to verify serum spermidine levels alongside the risk of CVD in the general population; (ii) we combined the data from the cross-sectional study and nested case-control study to increase the consistency and repeatability of our results, moreover, all study participants were from a socioeconomically homogeneous study population, reducing the possibility of bias and confounding factors in our data; (iii) we have detected the spermidine concentration in serum, rather than estimating the concentration from the diet, which allows a more accurate estimate of spermidine and stroke relationship. However, there are also several limitations of this study. First, our study cohort consisted only of adults selected from northeast rural areas of China, whose diversity may be limited. Therefore, we encourage careful testing in other general populations with greater diversity. Second, we were unable to further adjust for the confounding effects of dietary spermidine on the outcome because the data we collected did not initially contain information on dietary spermidine intake. Third, this study only focused on the association between baseline serum spermidine and stroke, however, disease occurrence is a dynamic process, thus, we encourage future studies to take into account the association between dynamic changes in serum spermidine and stroke.

In conclusion, we provide direct evidence for the association between higher serum spermidine levels and stoke incidence in a community-based general population. Our results may also provide a guide for the study of the pathogenesis of stroke. In the future, long-term exploration and reliable clinical trials may also be needed to confirm whether exogenous spermidine supplementation is beneficial to human health.

## Data Availability Statement

The raw data supporting the conclusions of this article will be made available by the authors, without undue reservation.

## Ethics Statement

The studies involving human participants were reviewed and approved by China Medical University Research Ethics Committee. The patients/participants provided their written informed consent to participate in this study.

## Author Contributions

YX, ZS, YS, DW, and LZ designed the study and analyzed the data. RZ, JX, and QX collected and analyzed the meteorological data. YX and ZS drafted the manuscript. YM, JZ, ZL, XG, GS, and FX contributed to the critical interpretation of the results and development of the report. YS, DW, and LZ proofread the manuscript. LZ financially supported this study. All authors contributed to the article and approved the submitted version.

## Conflict of Interest

The authors declare that the research was conducted in the absence of any commercial or financial relationships that could be construed as a potential conflict of interest.

## Publisher’s Note

All claims expressed in this article are solely those of the authors and do not necessarily represent those of their affiliated organizations, or those of the publisher, the editors and the reviewers. Any product that may be evaluated in this article, or claim that may be made by its manufacturer, is not guaranteed or endorsed by the publisher.

## Abbreviations

AUC: area under the curve
CAN: acetonitrile
CVD: cardiovascular disease
DALY: disability adjusted life years
CHD: coronary heart disease
DBP: diastolic blood pressure
FLD: fluorescence detection
HDL-C: high-density lipoprotein cholesterol
HPLC: high-performance liquid chromatography
HR: hazard ratio
LDL-C: low-density lipoprotein cholesterol
OD: odds ratio
ROC: receiver operator characteristic curve
SBP: systolic blood pressure
SD: standard deviation
SPD: spermidine trihydrochloride
TC: total cholesterol
TG: triglyceride.
